# Changes in Alzheimer's disease‐associated biomarkers in COVID‐19 patients presenting neurological symptoms

**DOI:** 10.1002/alz70855_106717

**Published:** 2025-12-24

**Authors:** Fernanda G. Q. Barros‐Aragão, Luis E. Santos, Talita Pinto, Thaís Lopes Pinheiro, Nathane Rezende, Fabiana Souza Lima, Bart Vanderborght, Gabriel Freitas, Guilherme B. de Freitas, Fernando A. Bozza, Andrea Silveira Souza, Erika Rodrigues, Carlos O Brandão, Paulo E. Mattos, Felipe K. Sudo, Fernanda Tovar‐Moll, Fernanda Guarino De Felice

**Affiliations:** ^1^ D'Or Institute for Research and Education, Rio de Janeiro, RJ, Brazil; ^2^ IDOR, Rio de Janeiro, RJ, Brazil; ^3^ Queen's University, Kingston, ON, Canada; ^4^ Oswaldo Cruz Foundation, Rio de Janeiro, RJ, Brazil; ^5^ D'Or Institute of Research and Education, Rio de Janeiro, RJ, Brazil; ^6^ Neurolife, Rio de Janeiro, RJ, Brazil; ^7^ D'Or Institute for Research and Education (IDOR), Rio de Janeiro, RJ, Brazil

## Abstract

**Background:**

COVID‐19 induces acute and long‐term neurological symptoms. Alzheimer's disease (AD) is a risk factor for severe COVID‐19, and COVID‐19 survivors may experience cognitive decline. Because inflammation plays a significant role in both diseases, links between COVID‐19 and AD have been hypothesized. However, it is unknown if COVID‐19 patients with neurological disturbance present molecular alterations related to AD pathology. Identifying possible molecular links between COVID‐19 and AD would improve patient follow‐up and late‐onset disease prevention. Here, we tested the possibility that COVID‐19 neurological patients present early AD‐related molecular neuropathological alterations.

**Method:**

In a retrospective analysis, we compared AD‐related cerebrospinal fluid (CSF) biomarkers of amyloid‐beta (Ab) proteinopathy (Ab42/40), tauopathy (phosphorylated Tau, pTau181), and total Tau from controls (*n* = 36), amnestic mild cognitive impairment (aMCI, *n* = 19), AD (*n* = 20), and COVID‐19 patients presenting important neurological alterations at hospitalization (*n* = 35). CSF biomarkers were correlated with systemic and central nervous system inflammation markers. In a prospective cohort (*n* = 41), we evaluated plasma Ab42, Ab40, and Tau longitudinal changes up to one‐year post‐COVID using SIMOA. Plasma biomarkers were correlated with cognitive outcomes. The Brazilian Ministry of Health and IDOR approved the study protocol.

**Result:**

We found that, at hospitalization, severe COVID‐19 patients with neurological symptoms presented elevated CSF Tau, like AD patients. However, we did not detect changes in CSF Ab42/Ab40, pTau‐181/Ab42, or Tau/Ab42 ratios. CSF pro‐inflammatory cytokine IL6 levels and AD‐related biomarkers (Tau, pTau181, Tau/Ab42, pTau‐181/Ab42) positivelycorrelated with systemic inflammatory index (SII). Up to one‐year post‐COVID, we found that plasma Tau/Ab42 selectively increased in patients with cognitive deficits. Plasma Tau longitudinal changes were most influenced by COVID‐19 disease severity (negatively) and SII (positively) at hospitalization and the presence of post‐COVID cognitive deficits (positively).

**Conclusion:**

Collectively, our findings put inflammation as a primary correlate of acute and persistent AD‐related molecular changes in COVID‐19 patients, urging careful follow‐up of COVID‐19 survivors with lingering inflammation or cognitive symptoms for possible risk of future AD.

